# Involvement of MAFB and MAFF in Retinoid-Mediated Suppression of Hepatocellular Carcinoma Invasion

**DOI:** 10.3390/ijms19051450

**Published:** 2018-05-13

**Authors:** Hiroyuki Tsuchiya, Seiya Oura

**Affiliations:** 1 Graduate School of Medicine, Tottori University, Tottori 680-8550, Japan; 2 Graduate School of Pharmaceutical Sciences, Osaka University, Osaka 565-0871, Japan; oura@biken.osaka-u.ac.jp

**Keywords:** retinoids, RARα, TFPI2, MAFB, MAFF, hepatocellular carcinoma, invasion

## Abstract

Retinoids exert antitumor effects through the retinoic acid receptor α (RARα). In the present study, we sought to identify the factors involved in the RARα-mediated transcriptional regulation of the tumor suppressor gene and the tissue factor pathway inhibitor 2 (TFPI2) in hepatocellular carcinoma (HCC). All-*trans*-retinoic acid (ATRA) was used in the in vitro experiments. Cell invasiveness was measured using trans-well invasion assay. ATRA significantly increased TFPI2 expression through RARα in a human HCC cell line known as HuH7. TFPI2 was vital in the ATRA-mediated suppression of HuH7 cell invasion. The musculo-aponeurotic fibrosarcoma oncogene homolog B (MAFB) significantly enhanced the activation of the TFPI2 promoter via RARα while MAFF inhibited it. The knockdown of RARα or MAFB counteracted the ATRA-mediated suppression of HuH7 cell invasion while the knockdown of MAFF inhibited the invasion. TFPI2 expression in HCC tissues was significantly downregulated possibly due to the decreased expression of RARβ and MAFB. Patients with HCC expressing low MAFB and high MAFF levels showed the shortest disease-free survival time. These results suggest that MAFB and MAFF play critical roles in the antitumor effects of retinoids by regulating the expression of retinoid target genes such as TFPI2 and can be promising for developing therapies to combat HCC invasion.

## 1. Introduction

Preclinical studies have shown that retinoids possess anticancer effects against different cancer cell lines in vitro [1,2,3]. Moreover, it was also demonstrated that retinoids reduced cancer risk by 70% in animal models [4,5]. The retinoic acid receptor α (RARα) is a central mediator of pleiotropic actions of retinoids. Many other factors also take part in retinoid action by modulating the transactivating functions of RARα. For example, a nuclear receptor co-repressor and a short heterodimer partner bind to RARα and suppress its function [6,7] while SP1 and the B-cell translocation gene 1 activate RARα via direct interaction [8,9]. Since retinoids produce severe side effects including tetracarcinogenesis, mucocutaneous cytotoxicity, and hypertriglyceridemia [10], elucidating the mechanism underlying retinoid-facilitated transcriptional regulation is crucial in developing next-generation retinoid-acting drugs with an improved specificity.

The tissue factor pathway inhibitor 2 (TFPI2) is a serine protease inhibitor that targets the factor VIIa–tissue factor complex as well as trypsin and plasmin [11,12]. TFPI2 indirectly inhibits the activation of the matrix metalloprotease (MMP), which needs trypsin and plasmin and suppresses cancer invasion [13,14,15,16]. This antitumor effect is supported by the fact that TFPI2 is silenced in various tumors including hepatocellular carcinoma (HCC) [15,16]. Therefore, agents with the ability to restore TFPI2 expression in tumors could be promising for developing novel antitumor drugs. Moreover, clarifying the mechanism underlying TFPI2 transcriptional regulation may be helpful in furthering our understanding of tumor development and progression.

In the present study, we aimed to investigate the effect of retinoids on TFPI2 expression and its regulatory mechanism. In addition, we asked whether TFPI2 is involved in retinoid-induced suppression of cancer cell invasion. Finally, it was addressed whether TFPI2 and factors involved in TFPI2 expression affect the prognosis of patients with HCC.

## 2. Results

### 2.1. TFPI2 Is Transcriptionally Regulated by ATRA

**Figure 1:**
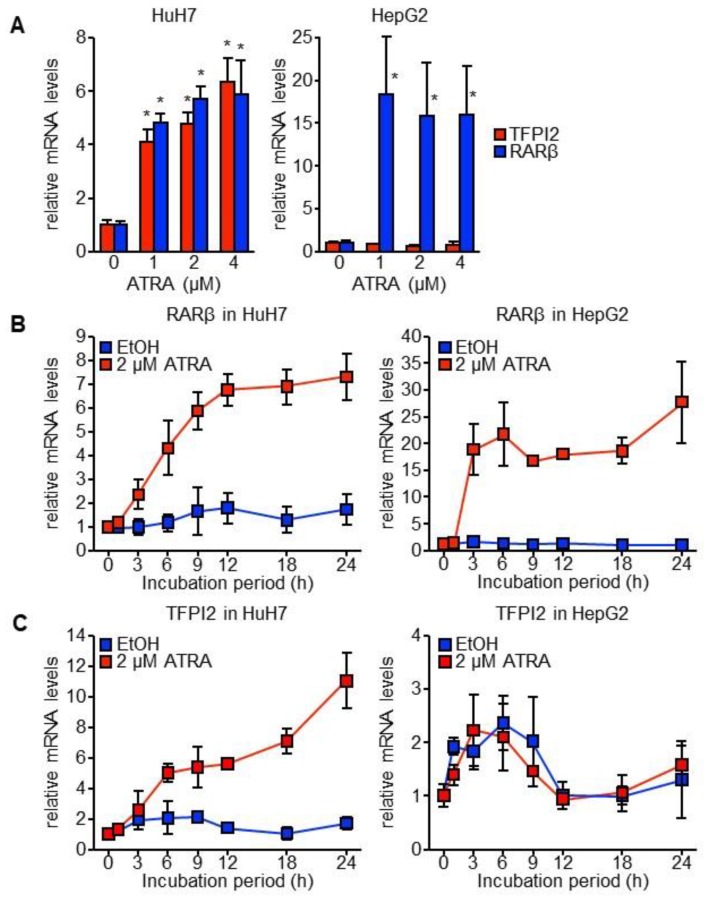
ATRA-induced TFPI2 expression in HuH7 cells. (**A**) Dose-dependent expression of TFPI2 (red) and RARβ (blue) mRNA in HuH7 (**left**) and HepG2 (**right**) cells following incubation with ATRA for 12 h (*n* = 4). * *p* < 0.05 (vs. 0 µM ATRA; Dunnett’s test), (**B**,**C**) Time courses of RARβ (**B**) and TFPI2 (**C**) mRNA expression in HuH7 (left) and HepG2 (right) cells following incubation with EtOH (blue) and 2 µM ATRA (red) for the indicated periods of time (*n* = 4).

Both HuH7 and HepG2 cell lines are derived from highly differentiated human HCC and are functionally similar in many aspects [17]. The mRNA levels of RARβ, which is a typical retinoid target gene, increased owing to ATRA in both the cell lines in a time-dependent and dose-dependent manner (see Figure 1A,B). However, ATRA increased TFPI2 mRNA levels only in HuH7 cells and not in HepG2 cells (see Figure 1A,C). Note that the *TFPI2* gene expresses three transcript variants. The primers for TFPI2 were designed to detect all three variants.

**Figure 2:**
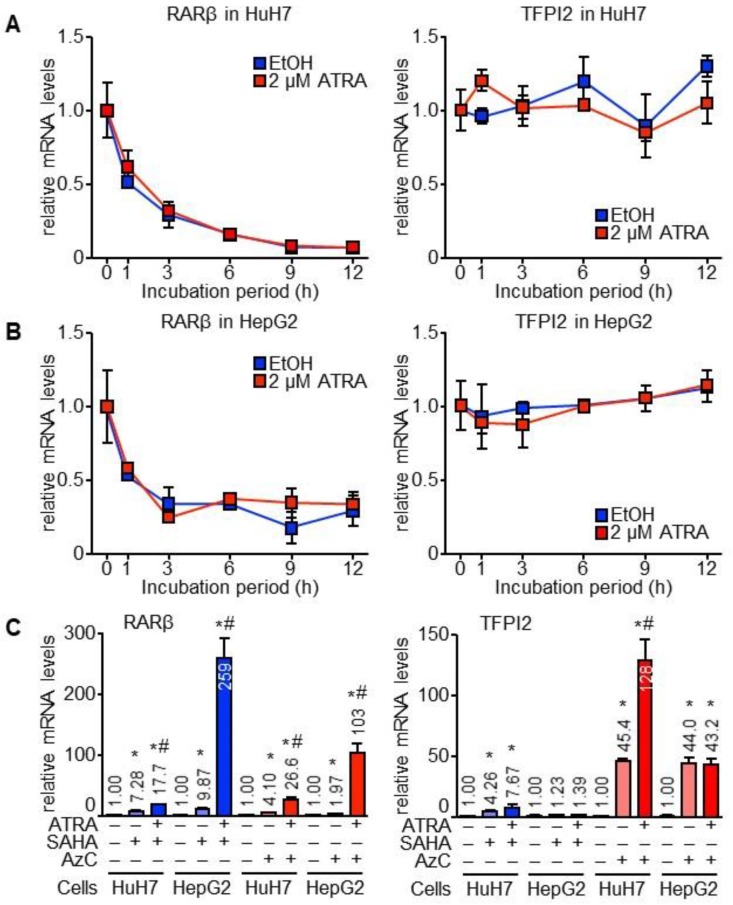
Transcriptional regulation of TFPI2 expression by ATRA in HuH7 cells. (**A**,**B**) HuH7 (**A**) and HepG2 (**B**) cells pre-treated with ActD incubated with EtOH (blue) and 2 µM ATRA (red) for the indicated times. Left, RARβ, right, TFPI2 (*n* = 4); (**C**) HuH7 and HepG2 cells pre-treated with *N*-hydroxy-*N*′-phenyloctanediamide (SAHA) (blue) and AzC (red) incubated with EtOH and 2 µM ATRA for 12 h. RARβ (**Left**), TFPI2 (**right**) (*n* = 4). * *p* < 0.05 (vs. non-treatment control), # *p* < 0.05 (EtOH vs. 2 µM ATRA in the presence of SAHA or AzC) (Tukey–Kramer’s test).

The RNA polymerase inhibitor actinomycin D (ActD) was added to the cells with ATRA to investigate whether ATRA increased the stability of TFPI2 mRNA. TFPI2 mRNA was more stable than RARβ mRNA in both of the HCC cell lines (see Figure 2A,B). However, the increase in TFPI2 mRNA levels due to ATRA in HuH7 cells was abolished by ActD (see Figure 2A), which suggests that ATRA transcriptionally regulates TFPI2 expression.

5-Aza-2′-deoxycytidine (AzC) and *N*-hydroxy-*N*′-phenyloctanediamide (SAHA) are inhibitors of DNA methyltransferase and histone deacetylase, respectively. These reagents significantly upregulated RARβ expression in both HuH7 and HepG2 cells, which was further boosted by ATRA (see Figure 2C). AzC instead of SAHA induced TFPI2 expression in HepG2, which suggested that epigenetic suppression by DNA methylation was released in the cells. Despite that, ATRA failed to influence TFPI2 expression (see Figure 2C). These results suggest that ATRA-induced TFPI2 expression is independent of the epigenetic mechanisms.

### 2.2. Through TFPI2, ATRA Suppresses HuH7 Cell Invasion

**Figure 3:**
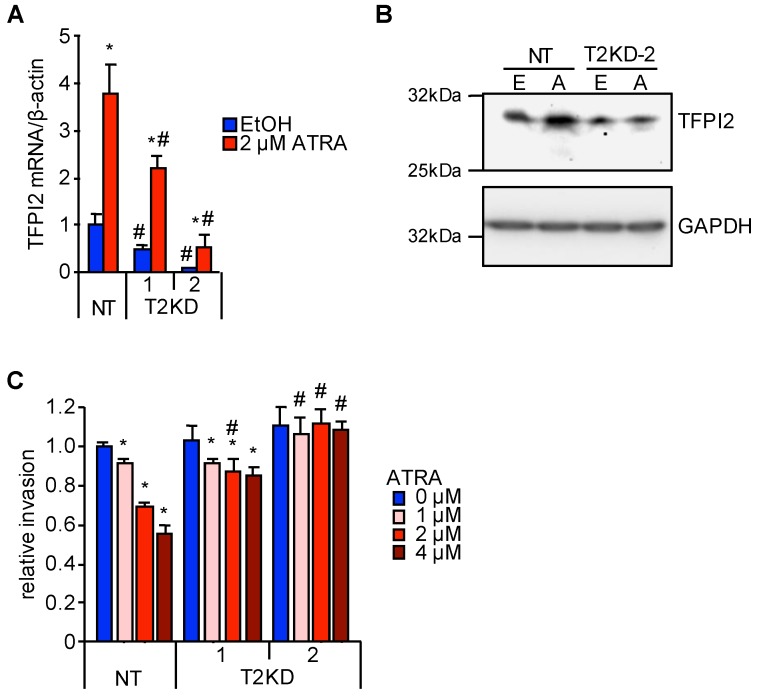
Abrogation of the suppressive effect of ATRA on HuH7 cell invasion by TFPI2 knockdown. (**A**) TFPI2 mRNA expression in HuH7 cells stably transfected with shNT, shTFPI2-1, and shTFPI2-2 (NT, T2KD-1, and T2KD-2 cells, respectively). The cells were treated with EtOH (blue) and 2 µM ATRA (red) for 12 h (*n* = 4). * *p* < 0.05 (EtOH vs. 2 µM ATRA), # *p* < 0.05 (vs. shNT) (Tukey–Kramer’s test); (**B**) TFPI2 expression in NT and T2KD-2 cells. The cells were treated with EtOH (E) and 2 µM ATRA (A) for 48 h. Glyceraldehyde-3-phosphate dehydrogenase (GAPDH) was shown as the loading control (**C**) Invasion capability of NT, T2KD-1, and T2KD-2 cells. The cells were treated with 0 µM (blue), 1 µM (light red), 2 µM (red), and 4 µM (dark red) of ATRA for 48 h (*n* = 6). * *p* < 0.05 (EtOH vs. 2 µM ATRA), # *p* < 0.05 (vs. shNT) (Tukey–Kramer’s test).

To investigate the biological significance of ATRA-induced TFPI2 expression, TFPI2 was stably knocked down in HuH7 cells using expression vectors of short hairpin RNA (shRNA) targeting TFPI2 (shTFPI2-1 and shTFPI2-2). We also created a control clone transfected with non-targeting shRNA (shNT). These cells were referred to as T2KD-1, T2KD-2, and NT cells, respectively. Although ATRA-induced TFPI2 expression was observed in T2KD-1 and T2KD-2 cells, it was decreased by approximately 50% and 15%, respectively, when compared with that in NT cells (see Figure 3A). The expression of the TFPI2 protein in T2KD-2 cells was also decreased when compared with NT cells and it is upregulated by ATRA, which was observed in NT cells. This was completely abolished in T2KD-2 cells (see Figure 3B). Treatment with ATRA for 48 h significantly suppressed the invasion of NT cells in a dose-dependent manner while this suppression with respect to T2KD-1 and T2KD-2 cells was significantly impaired, which correlated with the knockdown efficiency (see Figure 3C). Although TFPI2 is responsible for the ATRA-mediated inhibition of HuH7 cell invasion, ATRA also suppressed HepG2 cell invasion without inducing TFPI2 (see Figure 1A and Figure S1), which suggested that ATRA employs several mechanisms to suppress cancer invasion in the context of cell lines.

We then performed the microarray analysis to assess the expression profiles of NT and T2KD-2 cells in the presence or absence of 2 µM ATRA for 12 and 36 h. In total, 2061 probes and 961 probes with 1.5-fold or more changes in shTFPI2-2 at 12 and 36 h, respectively, were clustered into two groups (Cluster A, shNT > shTFPI2-2 at 12 h, Cluster B, shNT < shTFPI2-2 at 12 h, Cluster C, shNT > shTFPI2-2, 36 h, Cluster D, shNT < shTFPI2-2, 36 h) (see Figure S2). This cluster analysis also showed that changes in gene expression induced by ATRA were not markedly different for shNT and shTFPI2-2, which indicated that TFPI2 is a downstream factor of the retinoid signaling. Kyoto Encyclopedia of Genes and Genomes (KEGG) pathway analysis of the clustered genes showed that TFPI2 may be involved in the pathways related to cellular motility (hsa04151, hsa04360, and hsa04510), coagulation (hsa04610), nutritional metabolism (hsa00430, hsa01230, hsa01100, hsa00010, hsa00051, hsa01230, and hsa03320), and xenometabolism (hsa00980, hsa00860, hsa00983, hsa05034, and hsa05204) (see Figure S2).

### 2.3. MAFB and MAFF Modulate the Transactivation Activity of RARα on Human TFPI2 Promoter

**Figure 4:**
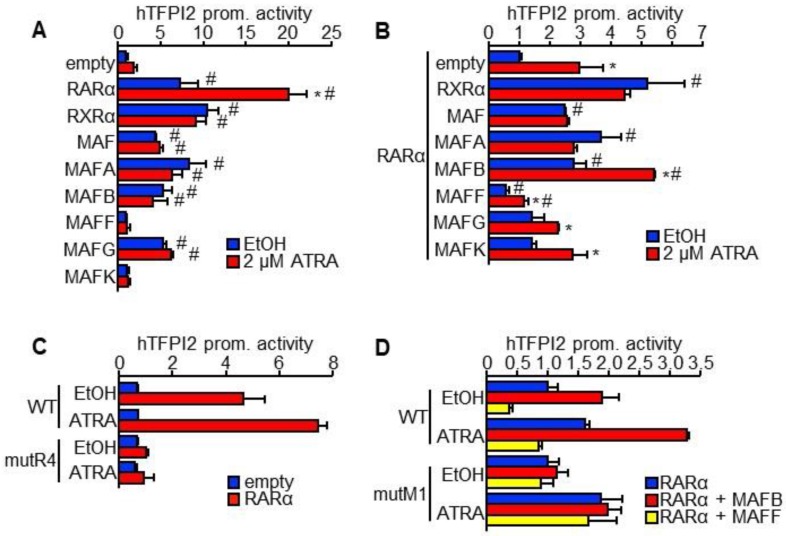
Regulation of human TFPI2 promoter by RARα, MAFB, and MAFF. (**A**,**B**) A luciferase reporter vector driven by the human TFPI2 promoter was transfected along with pDNA expressing the indicated transcription factor genes with (**B**) and without (**A**) RARα-pDNA into HuH7 cells (*n* = 4). Twenty-four hours after transfection, EtOH (blue) and 2 µM ATRA (red) were added to the cells, and further incubated for 24 h, which was followed by dual luciferase assay. * *p* < 0.05 (EtOH vs. 2 µM ATRA), # *p* < 0.05 (vs. empty) (Tukey–Kramer’s test), (**C**) Luciferase reporter vectors driven by wild-type (WT) and mutant half-site (mutR4) TFPI2 promoters were transfected with empty (blue) and RARα-expressing (red) pDNAs into HuH7 cells (*n* = 4). Twenty-four hours after transfection, EtOH and 2 µM ATRA were added to the cells and further incubated for 24 h, which was followed by the dual luciferase assay. (**D**) Reporter vectors driven by WT or mutant putative MAF binding site (mutM1) TFPI2 promoters were transfected with pDNAs expressing RARα (blue), RARα and MAFB (red), or RARα and MAFF (yellow) into HuH7 cells (*n* = 4).

Genome-wide binding analysis of musculoaponeurotic fibrosarcoma (MAF) F and MAFK in HepG2 cells from the ENCODE project [18] showed their binding sites in the region around the transcriptional start site of the human TFPI2 (see Figure S3A). This region was inserted upstream of the luciferase gene for reporter assays. RARα, RXRα, MAF, MAFA, MAFB, and MAFG significantly increased the promoter activity while MAFF and MAFK showed no effect (see Figure 4A). Only RARα showed enhanced transactivation activity in response to ATRA (see Figure 4A). Co-transfection with RXRα, MAF, MAFA, and MAFB enhanced the transactivation activity of RARα while MAFF was suppressed (see Figure 4B). Only MAFB maintained the responsiveness of RARα to ATRA while the remaining transcription factors abrogated it (see Figure 4B). Therefore, we chose to focus on investigating the effects of MAFB and MAFF on TFPI2 promoter activity. MAFB enhanced the transactivation activity of RXRα only in the presence of ATRA while MAFF showed no effect (see Figure S4A). The effects of MAFB and MAFF on the RXRα/RARα heterodimer were similar to those on RARα (see Figure S4A). Co-transfecting MAFB and MAFF showed that the transactivation activity of MAFB remained unaffected by MAFF (see Figure S4B).

In general, RARα binds to a tandem repeat of the consensus sequence 5′-RGKTCA-3′, which is termed the half-site [19]. However, the repeat sequence was not found in the promoter sequence of human TFPI2. Instead, four half-sites were present (see Figure S3A). In addition, two MAF-binding sites have been predicted by the ORCA toolkit [20]. Therefore, mutations were introduced into each of the four half-sites (mutR1–4) and two putative MAF-biding sites (mutM1–2) (see Figure S3A). The effects of RARα and MAFB/MAFF on the TFPI2 promoter were, respectively, eliminated by mutR4 and mutM1 even in the presence of ATRA (see Figure 4C,D, Figure S4C,D). These results helped us identify the MAF recognition element (MARE) and retinoic acid response element (RARE) at +6 and +66 relative to the transcription start site, respectively (see Figure S3B). Furthermore, these MARE and RARE were highly conserved among mammalian genomes (see Figure S3B). Binding of RARα with MAFB or MAFF was not observed under our experimental conditions (see Figure S5). Therefore, MAFB and MAFF might indirectly interact with RARα on the TFPI2 promoter.

### 2.4. MAFB, MAFF, and RARα Regulate TFPI2 Expression in HuH7 Cells and Their Invasion

**Figure 5:**
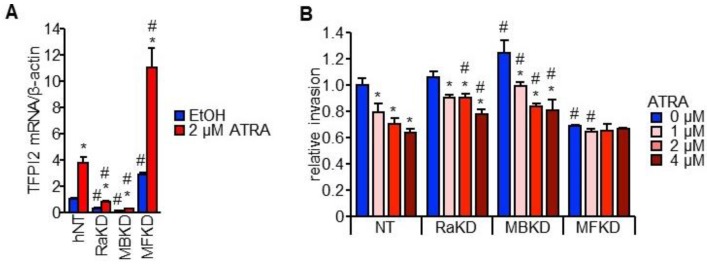
Effect of RARα, MAFB, and MAFF on HuH7 cell invasion through TFPI2. (**A**) TFPI2 mRNA expression in HuH7 cells stably transfected with shNT, shRARα, shMAFB, or shMAFF (NT, RaKD, MBKD, and MFKD cells, respectively). The cells were treated with EtOH (blue) or 2 µM ATRA (red) for 12 h (*n* = 4). * *p* < 0.05 (EtOH vs. 2 µM ATRA), # *p* < 0.05 (vs. shNT) (Tukey–Kramer’s test); (**B**) Invasion capability of HuH7 cells stably transfected with shNT, shRARα, shMAFB, or shMAFF. The cells were treated with 0 µM (blue), 1 µM (light red), 2 µM (red), and 4 µM (dark red) of ATRA for 48 h (*n* = 8). * *p* < 0.05 (vs. 0 µM ATRA), # *p* < 0.05 (vs. shNT) (Tukey–Kramer’s test).

HuH7 cells with stably knocked down RARα and MAFB (RaKD and MBKD cells, respectively) showed a decreased expression of TFPI2 while those with knocked down MAFF (MFKD) showed an increased TFPI2 expression (see Figure 5A and Figure S6A). Furthermore, HuH7 cells stably overexpressing MAFB (MBOE) showed a marked increase in TFPI2 expression while those overexpressing MAFF (MFOE) showed a decrease (see Figure S6B). ATRA-mediated suppression of cell invasion was significantly impaired by shRARα and shMAFB while MFKD cells showed a marked reduction in cell invasion irrespective of ATRA (see Figure 5B).

### 2.5. RARα and MAFB Contribute to the Downregulation of TFPI2 in HCC

**Figure 6:**
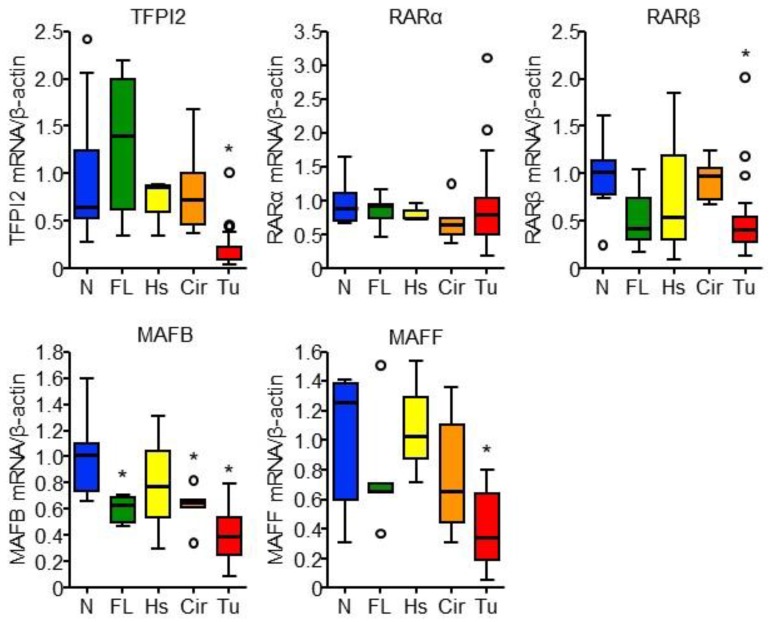
Gene expression in different types of liver diseases. Box and whisker plots of relative gene expression levels of TFPI2, RARα, RARβ, MAFB, and MAFF to β-actin in normal (N, *n* = 8; blue), fatty (FL, *n* = 5; green), hepatitis (Hs, *n* = 3; yellow), cirrhotic (Cir, *n* = 5; orange), and hepatocellular carcinoma (Tu, *n* = 23; red) liver tissues. Boxes indicate first and third quartiles and median values and whiskers represent 5 and 95 percentiles. Open circulars indicate outliers. * *p* < 0.05 (vs. normal liver tissues) (Dunnett’s test).

We checked whether the factors involved in TFPI2 transcription plays a role in human HCC. We performed quantitative PCR with 44 cDNA samples derived from eight normal, five fatty, three hepatitis, five cirrhotic, and 23 HCC liver tissues (see Table S1). The expression of TFPI2, RARβ, MAFB, and MAFF was significantly lower in HCC than that in normal liver tissue (see Figure 6). MAFB expression was also downregulated in fatty and cirrhotic liver tissues. The role of epigenetic mechanisms aside, the downregulation of TFPI2 can be attributable to the decreased expression of RARβ and MAFB in HCC. Correlation analysis demonstrated that the mRNA expression of TFPI2 in the clinical samples was significantly correlated with those of MAFB and MAFF (see Figure S7). Taking into account the observation that MAFF did not affect MAFB-induced activation of the TFPI2 promoter (see Figure S4B), MAFB may play a more important role in hepatic TFPI2 expression than the other transcription factors. Furthermore, MAFB and RARα expression in HCC was significantly downregulated as the TNM stage and histological grade increased while no correlation between TFPI2 and tumor grades was observed (see Table S2).

### 2.6. Involvement of MAFB, MAFF, and TFPI2 in the Progression and Prognosis of HCC

**Figure 7:**
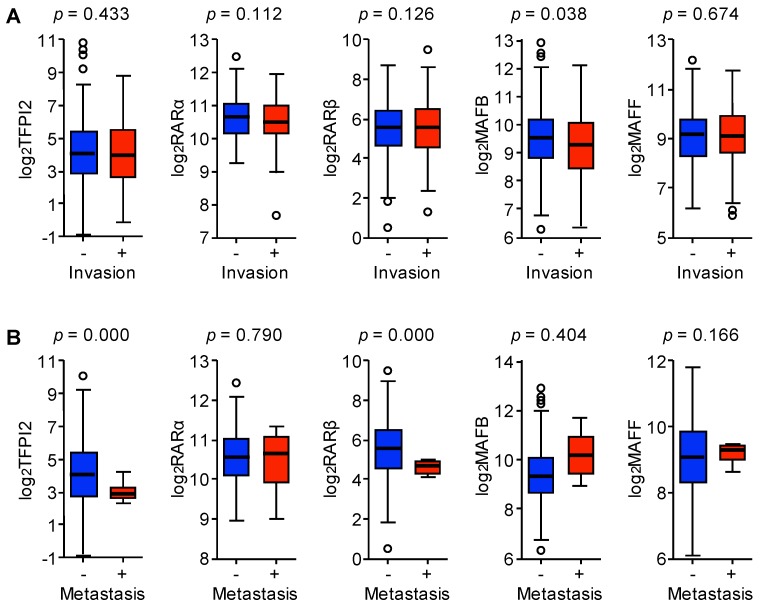
Correlation of gene expression in the livers of patients with HCC with vascular invasion and metastasis from the TCGA cohort. (**A**) Box and whisker plots of log 2 gene expression levels in HCC livers with (red) or without (blue) vascular invasion (TFPI2, *n* = 108 vs. 204, RARα, RARβ, MAFB, MAFF, *n* = 109 vs. 207). (**B**) Box and whisker plots of log 2 gene expression levels in HCC livers with (red) or without (blue) metastasis (*n* = 4 vs. 267). Boxes indicate first and third quartiles and median values and whiskers represent 5 and 95 percentiles. Open circulars indicate outliers. *p* values shown were calculated by the Welch’s *t*-test.

We further analyzed gene expression in patients with HCC by using the data generated by the TCGA Research [21]. HCC patients with vascular invasion showed significantly low MAFB expression while other genes remained unaffected (see Figure 7A). In addition, the expression of TFPI2 and RARβ was significantly low in patients with metastasis (see Figure 7B). This suggests that these genes may impact the prognosis of HCC patients.

**Figure 8:**
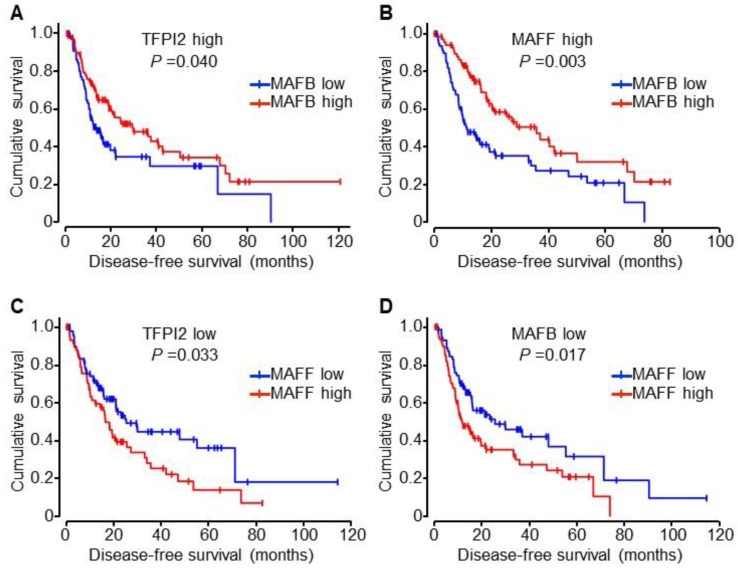
Kaplan–Meier analyses of disease-free survival of HCC patients from the TCGA cohort. (**A**,**B**) Disease-free survival curves of patients with lower (blue) or higher (red) MAFB expression in HCC with higher TFPI2 (*n* = 68 or *n* = 93, respectively) (**A**) or higher MAFF (*n* = 83 or 70, respectively) (**B**) groups, (**C**,**D**) Disease-free survival curves of patients with lower (blue) or higher (red) MAFF expression in HCC with lower TFPI2 (*n* = 94 or *n* = 62, respectively) (**C**) or lower MAFB (*n* = 73 or *n* = 83, respectively) (**D**) groups. *P* values shown were calculated by log-rank test. Median values of gene expression were used to divide the patients into the higher or lower groups.

Kaplan–Meier analysis revealed that the expression of TFPI2, RARα, RARβ, MAFB, and MAFF does not influence the disease-free survival (DFS) period (see Figure S8 [22]). However, patients with high MAFB expression in TFPI2 high or MAFF high groups showed longer DFS period than those with low MAFB expression (see Figure 8A,B and Figure S8B). In contrast, patients with high MAFF expression in TFPI2 low or MAFB low groups showed poorer DFS than those with low MAFF expression (see Figure 8C,D and Figure S8B). There was no significant difference among other groups including RARα and RARβ (see Figure S8B [22]).

### 2.7. Biological Significance of MAFB-Regulated ATRA Target Genes

The above findings in human HCC suggest anti-oncogenic properties of MAFB against MAFF. This prompted us to perform a microarray analysis by using the MAFB-knockdown and MAFF-knockdown HuH7 cells (MBKD and MFKD cells, Figure S6A). The genes showing expression that was 1.5-fold or higher due to ATRA in all cell lines were analyzed and clustered into six groups (see Figure S9). Clusters A and D contained genes whose induction ratio was increased and decreased by shMAFF, which implies that retinoid-target genes are regulated by MAFF (see Figure S9A). Clusters B and E, respectively, comprised retinoid-target genes that were positively and negatively regulated by MAFB (see Figure S9A). The genes in clusters C and F were, respectively, thought to be positively and negatively regulated by MAFB and MAFF. There was no cluster indicating genes regulated by MAFB and MAFF in an inverse manner (see Figure S9A).

The KEGG pathway analysis showed that the enriched pathways common to MAFB and MAFF were retinol metabolism (hsa0083) and the adipocytokine signaling pathway (hsa04920) (see Figure S9B). In addition, MAFB was thought to be involved in coagulation (hsa04610) and MAFF in the inflammatory response (hsa04668, hsa04064, and hsa04670) (see Figure S9B).

## 3. Discussion

MAF transcription factor family members play a vital role in various biological processes. They are divided into two groups based on structural characteristics including large MAFs, which involve c-MAF, MAFA, and MAFB, and small MAFs, which include MAFF, MAFG, and MAFK (see review [23]). Since small MAFs lack the transactivation domain, they can positively and negatively regulate transcription depending on their dimerization partners [24].

In the liver, small MAFs generally form a heterodimer with the Cap’n’Collar proteins to regulate cytoprotective genes related to xenobiotic metabolism, the oxidative stress response, and the redox homeostasis [24]. MAFG was recently revealed as a transcriptional repressor of bile acid synthesis and metabolism [25]. Although mice that lack only one of the small MAFs are viable and show mild phenotypic changes, knockout of all three small MAFs result in embryonic lethality with severe hypoplasia in the fetal liver due to increased apoptosis of hepatocytes and erythrocytes [26]. This suggests that despite being functionally redundant, small MAFs are crucial in proper liver development.

MAFB plays a role in the development of erythrocytes, pancreatic β cells, renal podocytes, and macrophages in the fetal liver [27,28,29]. The anti-oncogenic potential of MAFB is not entirely clear and tends to depend on the cellular context. Additionally, downregulation of MAFB expression is observed in neoplastic liver lesions induced by the activation of γ-aminobutyric acid A receptor in an *N*-diethylnitrosamine-induced hepatocarcinogenesis model [30]. By contrast, MAFB overexpression in hematopoietic stem/progenitor cells leads to the development of leukemia and myeloma [31]. Although MAFB transforms primary fibroblasts with an efficiency lower than that of c-MAF and MAFA [32], large MAFs do not transform chicken embryonic neuroretina cells but rather induce differentiation of crystallin-producing cells and counteract the proliferation of mutant RAS-transformed neuroretina cells [33]. In addition, MAFB induces the differentiation into macrophages of transformed hematopoietic precursor cells. c-MAF is thought to act as a tumor suppressor gene in prostate cancer [34,35].

We therefore hypothesized that MAFB confers its anti-oncogenic effects by antagonizing MAFF since MAFB-induced activation of the TFPI2 promoter remained unaffected by MAFF (see Figure S4B). Furthermore, the expression of MAFB in HCC tissues was significantly downregulated as the tumor stage increased (see Table S2). By contrast, the apparent difference in MAFB and MAFF expression was not observed in the presence or absence of vascular invasion or metastasis and there were no differences in DFS time based on individual gene expression. This might be attributable to the functional redundancy of large as well as small MAFs. However, HCC patients with lower MAFB and higher MAFF expression (median DFS time: 11.5 months) showed poorer DFS than those with higher expression of both MAFB and MAFF (35.8 months, *p* = 0.003) and those with lower expression of both MAFB and MAFF (25.3 months, *p* = 0.017) (see Figure 8B,D and Figure S8B). This suggests that MAFF functions as an oncogene in the absence of MAFB. Therefore, the complex interaction of MAFs must be considered when attempting to understand the physiological functions of MAFs in patients with HCC.

To our knowledge, this is the first report demonstrating the regulatory function of MAFB and MAFF in HCC cell invasion. TFPI2 is well known as a tumor suppressor gene and inhibits not only the factor VIIa/tissue factor complex but also plasmin, kallikrein, and trypsin [12]. By virtue of this property, TFPI2 also inhibits the activation of MMP1 and 3 by plasmin and trypsin [16]. However, Neaud et al. reported that TFPI2 showed a pro-invasive effect on HCC cell lines, which includes HepG2 and HuH7 cells [36]. They also demonstrated that the neutralization of factor VII with its specific antibody abolished the TFPI2-induced invasion of HCC cell lines [36]. The reason for this discrepancy is not clear at present. Our pathway analysis suggested that knockdown of TFPI2 in HuH7 cells influences the coagulation pathway (see cluster D in Figure S2). In addition, retinoid-target genes regulated by MAFB were also suggested to be classified into the coagulation pathway (clusters B & E in Figure S9). The modulation of clotting factor expression by ATRA may underlie the discrepancy effects of TFPI2 on cancer cell invasion.

Furthermore, the expression of TFPI2 is frequently silenced by promoter methylation in many tumor tissues including HCC [15,16,37,38]. Therefore, the restoration of TFPI2 expression suppresses the invasiveness of malignant tumors [16]. In contrast to the silencing mechanism, the transcription factors that are involved in TFPI2 transcription have not been well studied. In the present study, we identified the members belonging to the MAF transcription factor family as novel regulators of RARα transactivation activity in TFPI2 expression. We did not observe ATRA-induced TFPI2 expression in HepG2 cells (see Figure 1A). This may be explained by relatively low expression of MAFB in HepG2 cells when compared with HuH7 cells (see Figure S10A). The reporter assay in HepG2 cells demonstrated that RARα as well as MAFB and MAFF did not markedly affected TFPI2 promoter activity even in the presence of ATRA (see Figure S10B). However, when co-transfected MAFB or MAFF with RARα, MAFB significantly activated the promoter while MAFF suppressed it, which was observed in HuH7 cells (see Figure S10B). Therefore, it is implied that MAFB expression in HepG2 cells may not be sufficient for RARα to induce TFPI2 expression.

By dividing the HCC patients into groups with higher or lower TFPI2 expression, it was seen that in the higher TFPI2 group, those with higher MAFB expression showed better DFS than those with lower MAFB expression (29.3 months vs. 12.9 months, *p* = 0.040) while, in the lower TFPI2 group, those with lower MAFF showed better DFS than those with higher MAFF expression (25.3 months vs. 16.5 months, *p* = 0.033) (see Figure 7A,C and Figure S8B). These results suggest that MAFB and MAFF determine TFPI2 expression along with the epigenetic mechanisms. The limitations of our study include the inability to ascertain the correlation between RARα expression and HCC malignancy in combination with the expression of other genes. Low body retinoid stores are frequently observed in patients with chronic liver diseases including those with HCC [39,40]. It has also been shown that retinoid supplementation significantly improves the prognosis of patients with the hepatitis C virus-related HCC [41]. Therefore, it is likely that the activation of RARα by administration of retinoids possibly provides this benefit through TFPI2.

To summarize, TFPI2 is an anti-oncogene suppressing HCC invasion whose expression is regulated epigenetically and by retinoids. Moreover, MAFB and MAFF are novel regulators of RARα at least with respect to the transcription of TFPI2 (see Figure S11). This system may be implicated in the pathogenesis and progression of HCC and can be promising as a target for preventing and treating HCC in addition to predicting HCC prognosis.

## 4. Materials and Methods

### 4.1. Materials

The HuH7, HepG2, and KMST-6 cells, in addition to the plasmid DNAs (pDNAs) expressing human RXRα and MAFG, were purchased from the RIKEN BRC (Tsukuba, Japan) through the National Bio-Resource Project of the MEXT, Japan. The PDNA-expressing mouse Maf and those expressing mouse MafA or MafB were kindly provided by Hiroshi Nakajima (Graduate School of Medicine, Chiba University, Chiba, Japan) [42] and Satoru Takahashi (Faculty of Medicine, University of Tsukuba, Tsukuba, Japan) [43,44]. PDNAs expressing MAFF, MAFK, and RARα were obtained from NITE Biological Resource Center (Kisarazu, Japan). ShRNA expression vectors targeting TFPI2 (shTFPI2-1, TRCN0000072723; shTFPI2-2, TRCN0000072726), RARα (shRARα, TRCN0000020373), MAFB (shMAFB, TRCN0000017680), and MAFF (shMAFF, TRCN0000016448) along with pLKO.1-puro Non-Target shRNA (shNT) and fetal bovine serum (FBS), which were purchased from Sigma-Aldrich (St. Louis, MO, USA). Dual luciferase assay reagents and luciferase-expressing pDNAs were acquired from Promega (Madison, WI, USA). The cell invasion assay kit (medium basement membrane extract), QuikChange Lightning Site-Directed Mutagenesis Kit, and Viofectin transfection reagent were respectively purchased from Trevigen, Inc. (Gaithersburg, MD, USA), Agilent Technologies (Santa Clara, CA, USA), and Viogene (New Taipei City, Taiwan). ReverTra Ace, Thunderbird SYBR qPCR Mix, and KOD-Plus-Neo were purchased from Toyobo Co., Ltd. (Osaka, Japan). The Anti-Flag antibody and its magnetic agarose beads were purchased from MBL (Nagoya, Japan) and anti-Strep-tag II (ab76949) and anti-TFPI2 (ab186747) antibodies from Abcam (Cambridge, UK). ATRA and anti-GAPDH antibody (sc-365062) was purchased from Santa Cruz Biotechnology (Santa Cruz, CA, USA). Dulbecco’s modified Eagle’s medium (DMEM), ActD, ethanol (EtOH), puromycin, G418, and Sepasol-RNA I super G were purchased from Nacalai Tesque Inc. (Kyoto, Japan). AzC and SAHA were bought from Tokyo Chemical Industry Co., Ltd. (Tokyo, Japan).

### 4.2. Cell Culture and ATRA Treatments

HuH7 and HepG2 cells were cultured in DMEM supplemented with 10% FBS at 37 °C with 5% CO_2_. Four milliliters of ATRA stock solution was prepared with EtOH. When treating the cells with ATRA, the same volume of EtOH was added to control cells at a concentration of <0.1%.

### 4.3. Treatment with ActD, AzC, and SAHA

To suppress transcription, HuH7 and HepG2 cells were pre-treated with 5 µg/mL ActD for 30 min after which they were separately incubated with EtOH and 2 µM ATRA for 0 to 12 h. To check whether epigenetic mechanisms were involved, HuH7 and HepG2 cells were pre-treated with 2 µM SAHA for 36 h or with 2 µM AzC for 108 h after which the cells were separately incubated with EtOH and 2 µM ATRA for 12 h in the presence of SAHA or AzC.

### 4.4. Establishment of Transgenic Cell Lines

HuH7 cells were transfected with pDNAs of shNT, shTFPI2, shRARα, shMAFB, and shMAFF by Viofectin, which was followed by puromycin selection. HuH7 cells transfected with empty pDNA, pDNA expressing mouse MAFB, or human MAFF were selected by G418. Gene expression in isolated clones was analyzed by using reverse-transcription quantitative PCR.

### 4.5. Determination of mRNA Levels

The cells were lysed with sepazol and total RNA was purified according to the manufacturer’s instructions. Complementary DNA (cDNA) was synthesized with ReverTra Ace and was analyzed by quantitative PCR with Thunderbird SYBR qPCR Mix. The following PCR primers were used: β-actin, GATGCAGAAGGAGATCACTGC (sense) and TGATCCACATCTGCTGGAAG (anti-sense); TFPI2, GAACCTGTGATGCTTTCACC (sense) and TCCGGATTCTACTGGCAAAG (anti-sense); MAFB, TGAACTTTGCGCGTTAAGCC (sense) and TCCTTTCCTCGTTGCTCTCTTC (anti-sense); mouse MAFB, AGTCGTGCAGGTATAAACGC (sense) and GAGTTTCTCGCACTTGACCTTG (anti-sense); MAFF, ATCCCCTATCCAGCAAAGCTC (sense) and TTGAGCCGTGTCACCTCCTC (anti-sense); RARα, CATTGAGAAGGTGCGCAAAG (sense) and AGACACGTTGTTCTGAGCTG (anti-sense); RARB, GAAAAAGACGACCCAGCAAG (sense) and ATGAGAGGTGGCATTGATCC (anti-sense). Relative mRNA levels were measured using the 2^−ΔΔ*C*t^ method with β-actin as an internal control.

### 4.6. Cell Invasion Assay

Cells pre-cultured in serum-free DMEM for 24 h were seeded in top chambers containing a medium amount of basement membrane extract and were incubated in serum-free DMEM supplemented with 0–4 µM of ATRA for 48 h while the bottom chambers were filled with DMEM containing 10% FBS as well as the same concentration of ATRA. After the incubation, the cells remaining in the top chambers were discarded and invasive cells were recovered using a dissociation solution provided through the cell invasion assay kit. The recovered cells were stained with calcein AM and calcein fluorescence was measured at an excitation of 485 nm and an emission of 520 nm.

### 4.7. Dual Reporter Assay

To construct a human TFPI2 promoter reporter vector, PCR cloning was performed with LA Taq with GC buffer I (TaKaRa Bio, Otsu, Japan). In the reaction, genomic DNA isolated from a human immortalized the fibroblast cell line, KMST-6, was used as a template and the following oligoDNAs were used as primers: hTFPI2pro-For, CGTCCTCGAGGTCCACACAAAGCAGCTT; hTFPI2pro-Rev, GTCGAGATCTGGGCAAGGCGTCCGAGAAA. The PCR products were digested with XhoI and BglII, which was followed by ligation into the same restriction sites of pGL4.10 [luc2]. The promoter sequence was confirmed by DNA sequencing. Site-directed mutagenesis was performed according to the manufacturer’s instructions by using the following mutagenic primers: mutR1-For, GTGCGGAGTTCTTGGAAAAGAGCGGAGCGGGATTCG; mutR1-Rev, CGAATCCCGCTCCGCTCTTTTCCAAGAACTCCGCAC; mutR2-For, CCACCTCTTGAAGGCATGAAGAAAAGGTATTTGAAAGGCTGGTGG; mutR2-Rev, CCACCAGCCTTTCAAATACCTTTTCTTCATGCCTTCAAGAGGTGG; mutR3-For, TGGGAAATCTGCAAGCTAGACGGAAAAAGAATGCTAGTGATTGCGCTGTAAAGAAGC; mutR3-Rev, GCTTCTTTACAGCGCAATCACTAGCATTCTTTTTCCGTCTAGCTTGCAGATTTCCCA; mutR4-For, CCCCGCCAAGTTGAAAAGTTTTTCCTGCCTCCCAAACTTTCTC; mutR4-Rev, GAGAAAGTTTGGGAGGCAGGAAAAACTTTTCAACTTGGCGGGG; mutM1-For, CTAGCTTGCAGATTTCCCATTACTTTCCAAAACGCTCCCTCAGGGCG; mutM1-Rev, CGCCCTGAGGGAGCGTTTTGGAAAGTAATGGGAAATCTGCAAGCTAG; mutM2-For, GCGGGGTGACAGTCCCCGTGCATTTAATAGCCACCCCTCA; mutM2-Rev, TGAGGGGTGGCTATTAAATGCACGGGGACTGTCACCCCGC.

These reporter vectors and pDNAs expressing transcription factors were transfected into HuH7 cells using Viofectin. The dual luciferase assay was performed 48 h after transfection using pGL4.74[hRluc/TK] expressing renilla luciferase with the herpes simplex virus thymidine kinase promoter (Promega) acting as a transfection control.

### 4.8. Gene Expression Data in Clinical Samples

CDNAs derived from various liver tissues (TissueScan Liver Cancer Tissue qPCR Panel I) were purchased from Origene Technologies (Rockville, MD, USA). RNA-seq data of HCC tissues were obtained from the TCGA Research Network [21], which also provides DFS times, and the presence of vascular invasion or metastasis.

### 4.9. Transcription Profiling

MRNAs were recovered from cells treated with EtOH or 2 µM ATRA for 12 h by using sepazol and were purified with the RNeasy Plus Mini Kit (QIAGEN, Redwood City, CA, USA). The expression profile was analyzed by the Human HT-12 v4 Expression BeadChip (Illumina, San Diego, CA, USA) at Macrogen Japan (Kyoto, Japan). The microarray data were deposited in the ArrayExpress database (Accession No. E-MTAB-6447). Cluster analysis was performed using Cluster 3.0 and the results were visualized using Java Tree View. The Database for Annotation, Visualization, and Integrated Discovery online tool was used to enrich the Kyoto Encyclopedia of Genes and Genomes (KEGG) pathway.

### 4.10. Immunoprecipitation-Western Blotting

PDNA expressing Strep-tag-fused RARα was constructed as follows. Internal ribosome entry site of pIRES2 DsRed-Express2 (Clontech, Foster City, CA, USA) was amplified by KOD-Plus-Neo with the forward primer, GCGGGTCGACGCCCCTCTCCCTCCCC, and the reverse primer, GCGGGGATCCTGTGGCCATATTATCATCGT. The PCR product was digested with SalI and BamHI and was then ligated into the same restriction sites of pAcGFP-N1 (Clontech), which produced pIRES2-AcGFP. RARα cDNA was amplified by using pDNA expressing human RARα as a template with the forward primer, GGCGCTCGAGCACTGTTGGCCTACTGG, and the reverse primer, CGCCGAATTCTTACTTTTCAAACTGCGGATGGCTCCACGGGGAGTGGGTGGCCG, which fuses a Strep tag to the C-terminal of RARα. The PCR product was digested with XhoI and EcoRI and was then ligated into the same restriction sites of pIRES2-AcGFP. This resulted in a pDNA expressing Strep-tag-fused RARα.

PDNAs expressing Flag-tag-fused MafB and MAFF were constructed as follows. Mouse MafB and human MAFF cDNAs were amplified by KOD-Plus-Neo with the MafB forward primer, GGCGGCTAGCCACCATGGACTACAAGGATGACGATGACAAGATGGCCGCGGAGCTGAGC, which fuses a Flag tag to the N-terminal of MafB and the MafB reverse primer, CGCCGTCGACTCACAGAAAGAACTCAGGAGAG, in addition to the MAFF forward primer, GGCGGCTAGCCACCATGGACTACAAGGATGACGATGACAAGATGTCTGTGGATCCCCTATC, which fuses a Flag tag to the N-terminal of MAFF and the MAFF reverse primer, GGGGGTCGACTAGGAGCAGGAGGCCGGG. The PCR products were digested with NheI and SalI and were then ligated into the same restriction sites of pIRES2 DsRed-Express2, which result in pDNAs expressing Flag-tag-fused MAFB and MAFF.

The pDNA expressing Strep-tag-fused RARα was co-transfected into HEK293 cells with either empty pDNA or pDNAs expressing Flag-tag-fused MAFB or MAFF. Forty-two hours after transfection, EtOH and 2 µM ATRA were separately added to the cell mixture. Following a six hour incubation, the cells were lysed with NP40 buffer (1% NP40, 50 mM Tris-HCl [pH 8.0] and 150 mM NaCl). The lysates were incubated overnight with anti-Flag-tag antibody-conjugated magnetic beads at 4 °C. The whole-cell lysates and precipitates were separated by using SDS-PAGE and immunoblotting with anti-Strep-tag and anti-Flag-tag antibodies.

### 4.11. Statistical Analysis

Data were expressed as mean ± standard deviation. Statistical analysis was performed using SPSS (version 24, SPSS Inc., Chicago, IL, USA). Probability values less than 0.05 were considered statistically significant.

## Supplementary Materials

Supplementary materials can be found at http://www.mdpi.com/1422-0067/19/5/1450/s1.

## Abbreviations

RARα	retinoic acid receptor α
TFPI2	tissue factor pathway inhibitor 2
HCC	hepatocellular carcinoma
ATRA	all-*trans*-retinoic acid
MAFB	musculoaponeurotic fibrosarcoma oncogene homolog B
MAFF	musculoaponeurotic fibrosarcoma oncogene homolog F
ActD	actinomycin D
AzC	5-aza-2′-deoxycytidine
SAHA	*N*-hydroxy-*N*′-phenyloctanediamide
