# Supplementary material for: Involvement of MAFB and MAFF in Retinoid-Mediated Suppression of Hepatocellular Carcinoma Invasion
Source: Int J Mol Sci. 2018 May 13;19(5):1450. doi: 10.3390/ijms19051450 (PMC5983688; doi:10.3390/ijms19051450)
Supplement: Supplementary file 1 [file ijms-19-01450-s001.pdf]

Supplementary Materials: Involvement of MAFB and MAFF in Retinoid-Mediated Suppression of Hepatocellular Carcinoma Invasion

Hiroyuki Tsuchiya and Seiya Oura

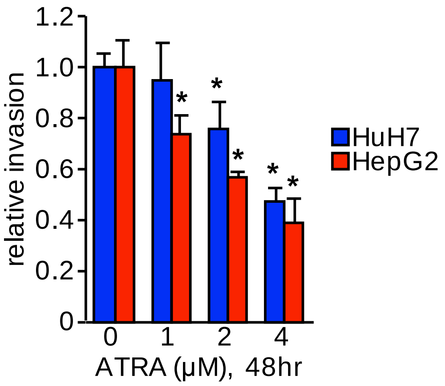

Figure S1. Suppressive effect of ATRA on HuH7 (blue) and HepG2 (red) cell invasion. HuH7 and HepG2 cells were treated with the indicated concentration of ATRA for 48 h (n = 6). *, p < 0.05 (0 μM vs. 2 μM ATRA) (Dunnett's test).

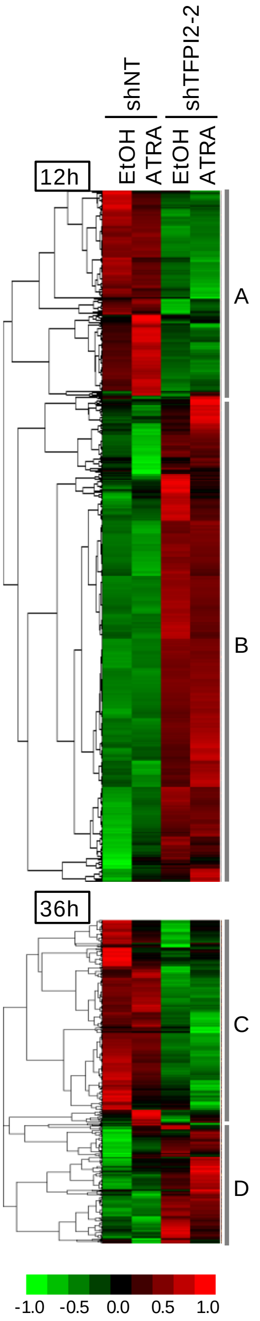

KEGG PATHWAY*	P-value	Fold Enrichment	FDR
**Cluster A (shNT > shTFPI2, 12 h)**			
PI3K-Akt signaling pathway (hsa04151)	0.002	2.31	2.24
Axon guidance (hsa04360)	0.024	2.79	26.34
Taurine and hypotaurine metabolism (hsa00430)	0.024	12.08	26.67
Focal adhesion (hsa04510)	0.042	2.15	41.82
**Cluster B (shNT < shTFPI2, 12 h)**			
Mineral absorption (hsa04978)	0.000	4.83	0.00
Lysosome (hsa04142)	0.001	2.49	0.66
Biosynthesis of amino acids (hsa01230)	0.002	2.79	2.62
Metabolic pathways (hsa01100)	0.005	1.28	6.47
Glycolysis / Gluconeogenesis (hsa00010)	0.008	2.61	10.60
Biosynthesis of antibiotics (hsa01130)	0.013	1.72	16.30
Fructose and mannose metabolism (hsa00051)	0.047	2.98	47.19
Antigen processing and presentation (hsa04612)	0.047	2.09	47.35
**Cluster C (shNT > shTFPI2, 36 h)**			
Steroid hormone biosynthesis (hsa00140)	0.003	4.79	3.91
Butanoate metabolism (hsa00650)	0.004	7.35	5.30
Metabolism of xenobiotics by cytochrome P450 (hsa00980)	0.010	3.76	12.37
Metabolic pathways (hsa01100)	0.011	1.42	13.28
Porphyrin and chlorophyll metabolism (hsa00860)	0.020	4.73	23.07
Drug metabolism - other enzymes (hsa00983)	0.027	4.32	29.92
Ascorbate and aldarate metabolism (hsa00053)	0.029	5.88	31.44
Taurine and hypotaurine metabolism (hsa00430)	0.030	10.83	31.90
Alcoholism (hsa05034)	0.033	2.24	35.14
Biosynthesis of amino acids (hsa01230)	0.037	3.22	38.62
**Cluster D (shNT < shTFPI2, 36 h)**			
Mineral absorption (hsa04978)	0.000	12.52	0.00
Complement and coagulation cascades (hsa04610)	0.001	5.84	1.38
PPAR signaling pathway (hsa03320)	0.006	5.16	6.78
Ribosome (hsa03010)	0.009	3.39	10.24
Biosynthesis of antibiotics (hsa01130)	0.029	2.44	30.55
Biosynthesis of amino acids (hsa01230)	0.038	3.89	37.83
Pertussis (hsa05133)	0.040	3.84	39.11
Chemical carcinogenesis (hsa05204)	0.049	3.60	45.56

*Analyzed by DAVID Bioinformatics Resources

Figure S2. KEGG pathway analysis of gene expression profile in TFPI2 knockdown cells. HuH7 cells stably transfected with shNT or shTFPI2-2 were treated with EtOH or 2 μM ATRA for 12 or 36 hr. MRNAs were recovered from the cells, and subjected to microarray analysis using Illumina Human HT-12 v4 Expression BeadChip (ArrayExpress accession, E-MTAB-6447).

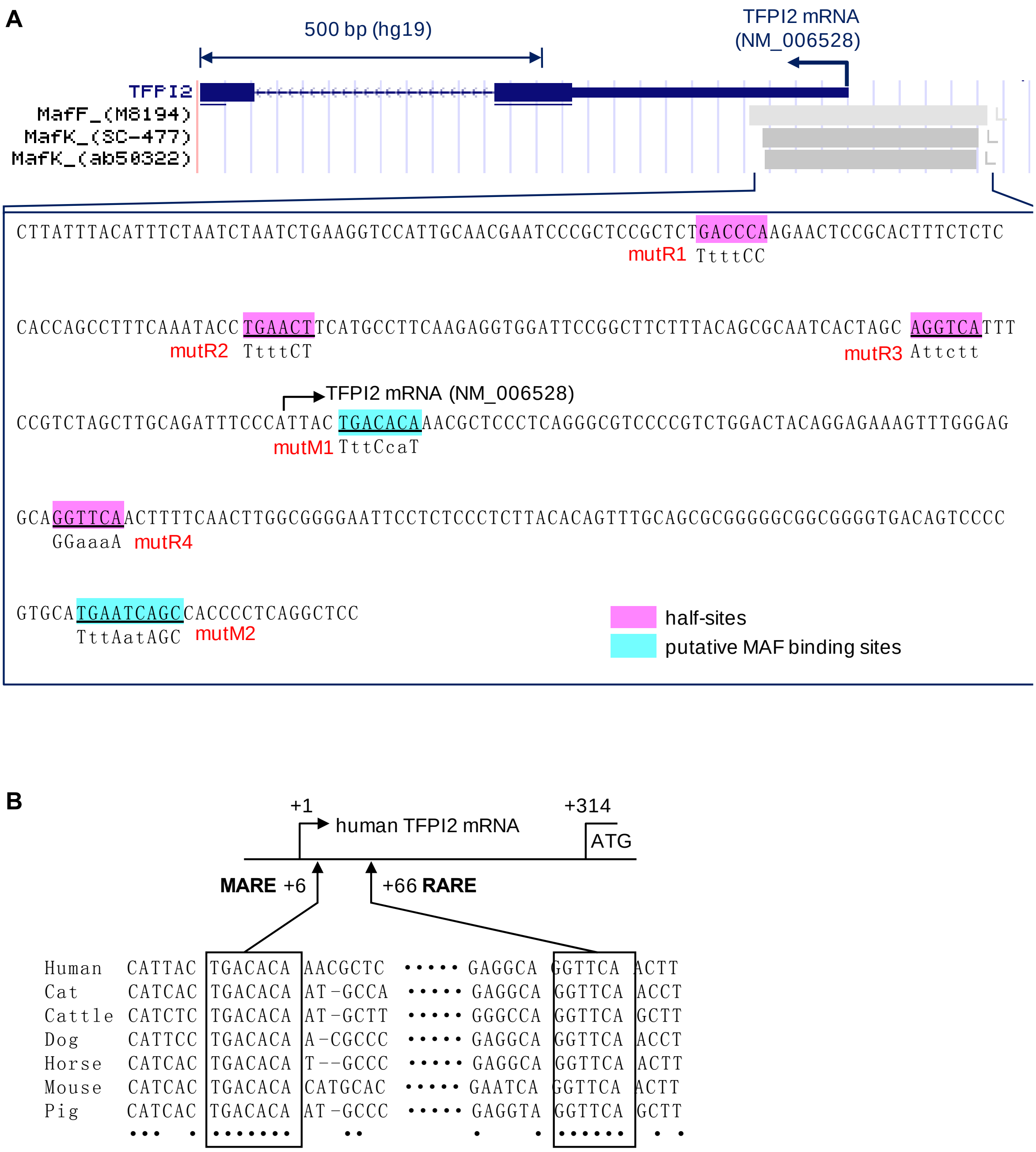

Figure S3. Promoter region of human TFPI2 gene. (**A**) Human genome map (GRCh37/hg19: chr7: 93,519,500–93,520,500) including the promoter region, the first exon, and a part of the second exon of TFPI2 gene was retrieved from the UCSC genome browser (https://genome.ucsc.edu/), together with MAFF (antibody: M8194) and MAFK (antibodies: SC-477, ab50322) ChIP-seq data from the ENCODE project (UCSC Accession: wgEncodeEH001841, wgEncodeEH001850, and wgEncodeEH001842, respectively). The sequence shown below the map is MAFF and MAFK binding region, where four RGKTCA (half-site) sequences (magenta) and two putative MAF binding sites (cyan) were found. Mutant sequences (mutR1-4, and mutM1-2) for reporter assay are also shown below the wild-type sequences; (**B**) Mammalian genome sequences from 5000 bp upstream to 3000 bp downstream of the transcription start site of TFPI2 mRNA (cat, XM_003982759; cattle, NM_182788; dog, XM_532462; horse, XM_001492769; mouse, NM_009364; pig, AK234618) were downloaded from the NCBI database, and aligned to that of human genome sequence (NM_006528) using the BLAST program (https://blast.ncbi.nlm.nih.gov/Blast.cgi).

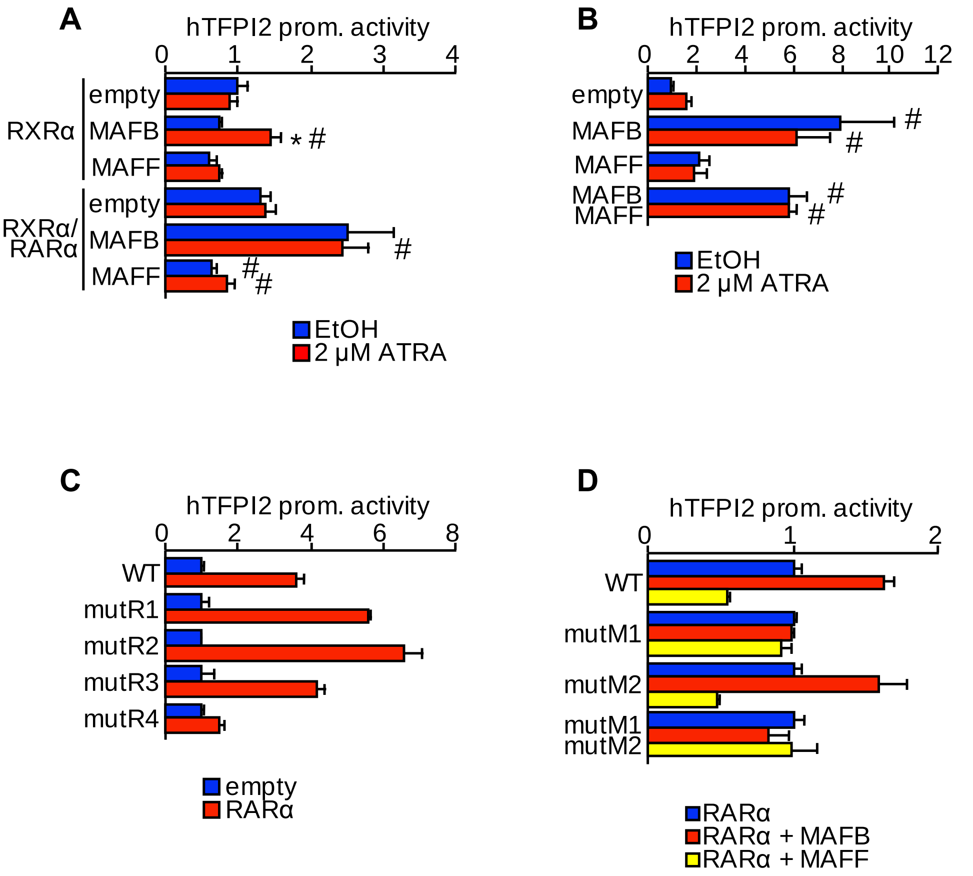

Figure S4. Identification of RARE and MARE in the human TFPI2 promoter. (**A**,**B**) A luciferase reporter vector driven by the human TFPI2 promoter was transfected along with pDNA expressing the indicated transcription factor genes into HuH7 cells. At 24 h post-transfection, EtOH (blue) or 2 μM ATRA (red) were added to the cells, and further incubated for 24 h, followed by dual luciferase assay ($n = 4$). *, $p < 0.05$ (EtOH vs. 2 μM ATRA); #, $p < 0.05$ (vs. empty) (Tukey-Kramer's test); (**C**) Reporter vectors driven by wild-type (WT), or mutant half-site (mutR1-4) TFPI2 promoters were transfected along with empty (blue) or RARα-expressing (red) pDNAs into HuH7 cells. The cells were incubated for 48 h, followed by dual luciferase assay ($n = 4$); (**D**) Reporter vectors driven by WT, or mutant putative MAF binding site (mutM1, 2 or both) TFPI2 promoters were transfected with pDNAs expressing RARα (blue), RARα and MAFB (red), or RARα and MAFF (yellow) into HuH7 cells ($n = 4$).

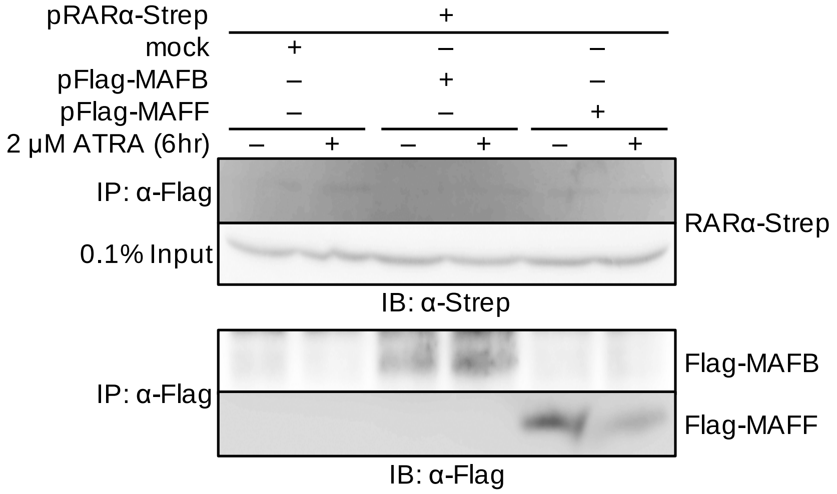

Figure S5. Assessment of direct interaction between RARα and, MAFB or MAFF. HEK293 cells co-transfected with pDNAs expressing Strep-tag-fused RARα, and Flag-tag-fused MAFB or MAFF were treated EtOH or 2 μM ATRA for 6 hr. The whole cell extracts (0.1% input) or immunoprecipitates by anti-Flag-antibody were subjected to immunoblotting with anti-Strep-tag or anti-Flag-tag antibodies.

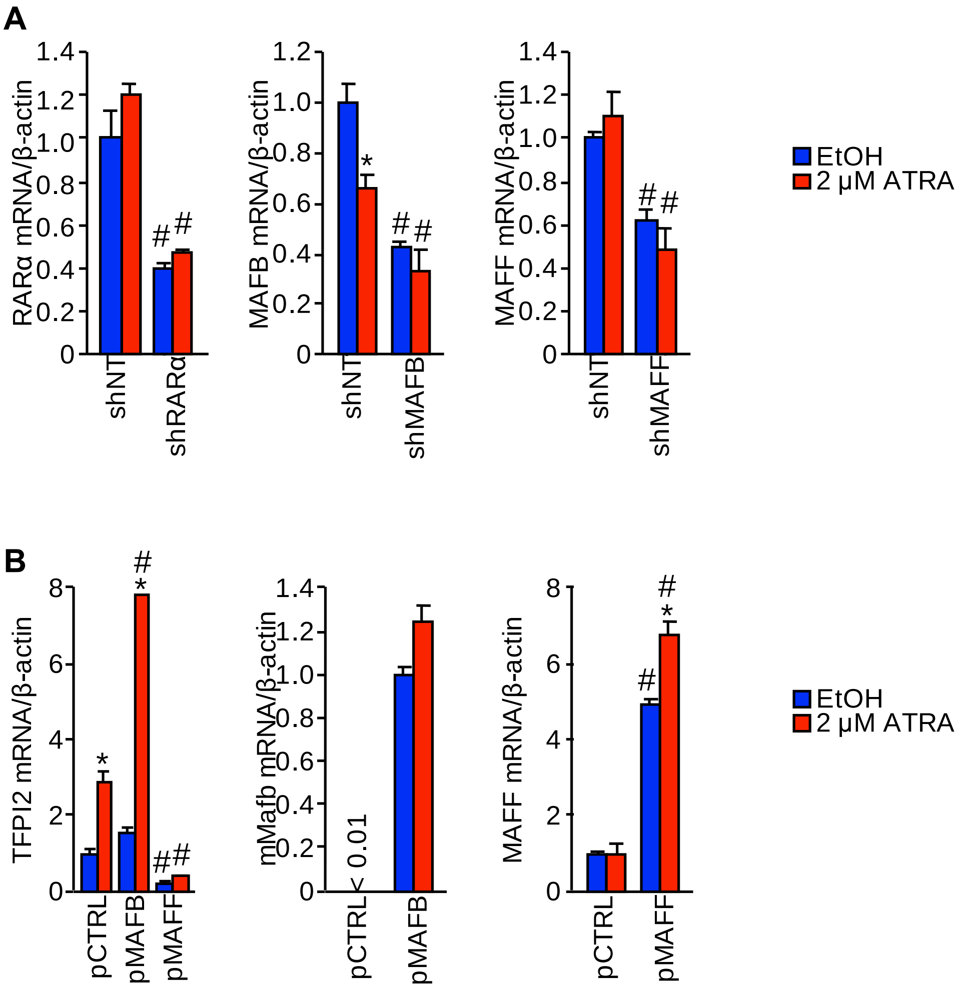

Figure S6. Evaluation of knockdown and overexpression efficiencies of RAR*α*, MAFB, and MAFF. (**A**) RAR*α*, MAFB, or MAFF expression in HuH7 cells stably transfected with shNT, shRAR*α*, shMAFB, or shMAFF. The cells were treated with EtOH (blue) or 2 μM ATRA (red) for 12 h ($n = 5$). *, $p < 0.05$ (EtOH vs. 2 μM ATRA); #, $p < 0.05$ (vs. shNT) (Tukey-Kramer's test); (**B**) Human TFPI2, mouse MafB, or human MAFF expression in HuH7 cells stably transfected with empty (pCTRL), mouse MafB (pMAFB), or human MAFF (pMAFF) expression vectors. The cells were treated with EtOH (blue) or 2 μM ATRA (red) for 12 h ($n = 4$). *, $p < 0.05$ (EtOH vs. 2 μM ATRA); #, $p < 0.05$ (vs. pCTRL) (Tukey-Kramer's test).

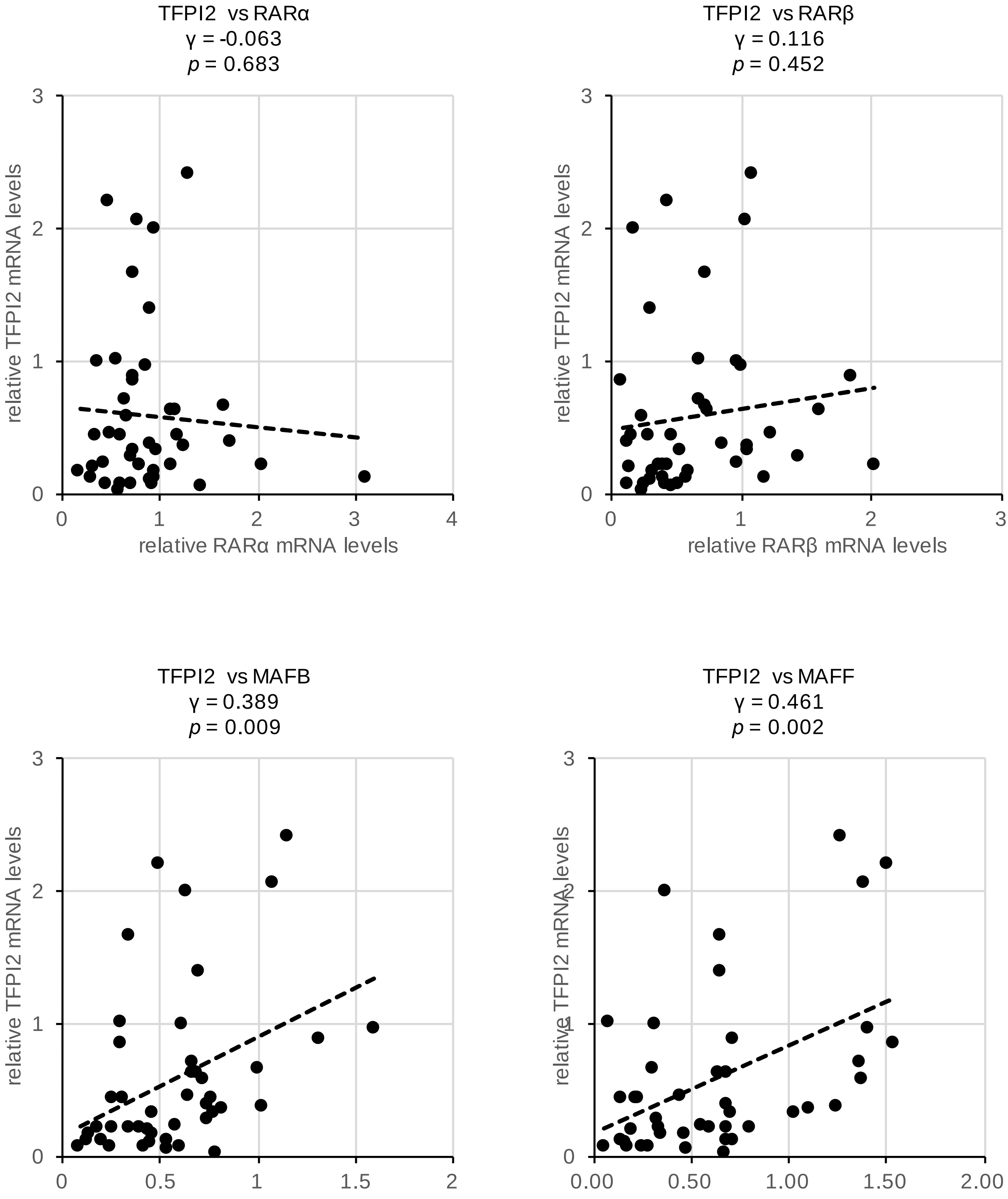

Figure S7. Correlation plots of TFPI2 mRNA expression levels with RARα, RARβ, MAFB and MAFF. Pearson correlation coefficients (γ) and *p* values are shown.

A

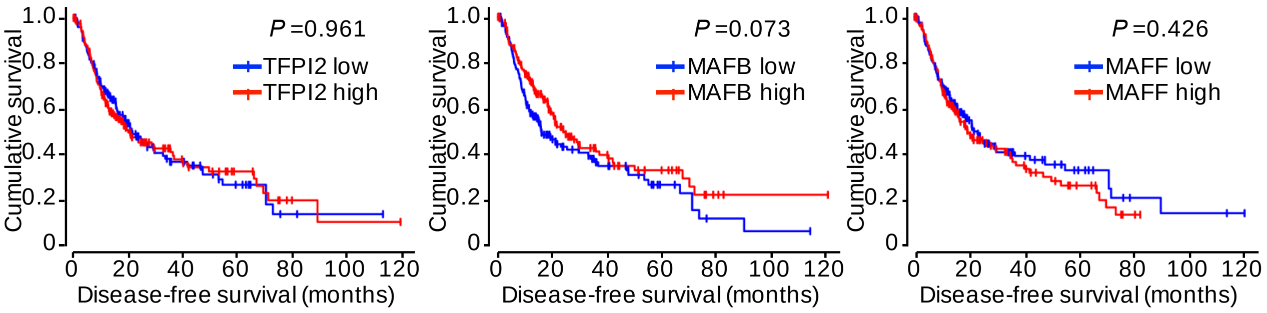

B

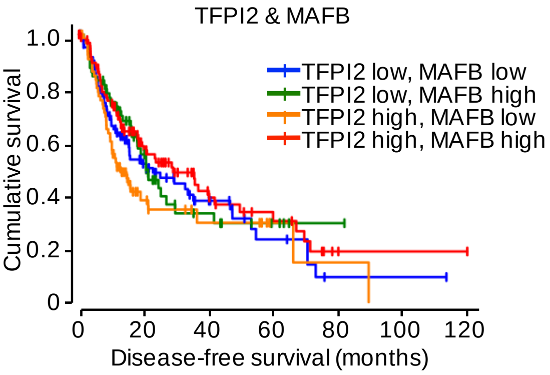

gene expression in HCC

TFPI2	MAFB	Median DFS Time (months (95% CI))	log-rank test
low	low	23.0 (9.7 – 36.5)	
	high	20.9 (15 – 27)	
high	low	12.9 (7.6 – 18.3)	0.040
	high	29.3 (14.6 – 44.1)	

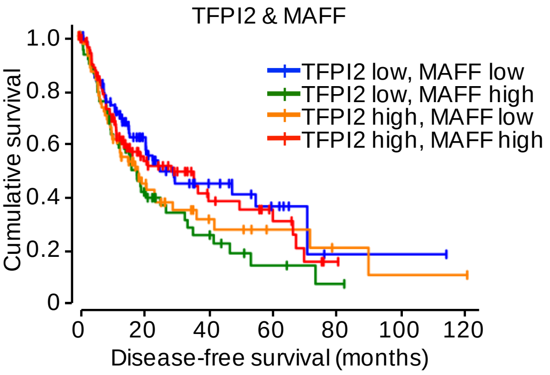

gene expression in HCC

TFPI2	MAFF	Median DFS Time (months (95% CI))	log-rank test
low	low	25.3 (15.9 – 34.8)	0.033
	high	16.5 (12.6 – 20.4)	
high	low	18.4 (11.3 – 25.7)	
	high	28.9 (11.2 – 46.7)	

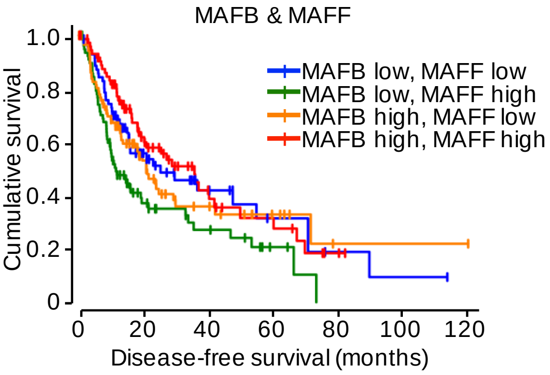

gene expression in HCC

MAFB	MAFF	Median DFS Time (months (95% CI))	log-rank test
low	low	25.3 (5.7 – 45)	0.017
	high	11.5 (6.7 – 16.4)	
high	low	20.9 (16 – 25.9)	0.003
	high	35.8 (22.5 – 49.3)	

Figure S8. Kaplan-Meier analyses of disease-free survival of HCC patients from the TCGA cohort. (**A**) Disease-free survival curves of patients with lower (blue) or higher (red) TFPI2 (n = 156 or 161, respectively; **left**), MAFB (n = 156 or 161, respectively; **middle**), and MAFF (n = 164 or 153, respectively; **right**) expression in HCC. (**B**) Disease-free survival curves of patients with HCC, and summary of estimated median disease-free survival (DFS) time (months) and 95% confidence interval (CI). The patients were divided based on the expression of TFPI2 and MAFB (**upper**; n = 88 (TFPI2 low/MAFB low; blue), 68 (low/high; green), 68 (high/low; orange), 99 (high/high; red)), TFPI2 and MAFF (**middle**; n = 94 (TFPI2 low/MAFF low; blue), 62 (low/high; green), 70 (high/low; orange), 97 (high/high; red)), or MAFB and MAFF (**lower**; n = 73 (MAFB low/MAFF low; blue), 83 (low/high; green), 91 (high/low; orange), 76 (high/high; red)). P values shown were calculated by log-rank test. Median values of gene expression levels were used to divide the patients into the higher or lower groups.

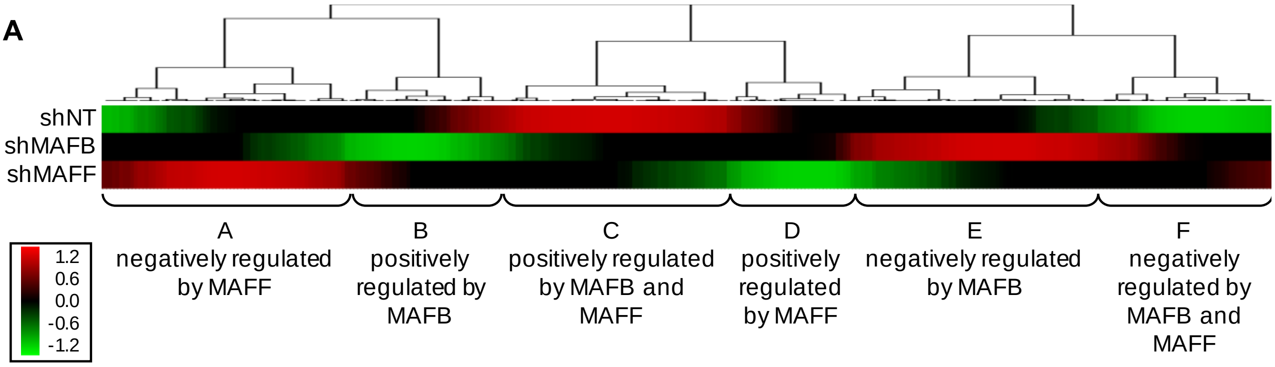

B

KEGG PATHWAY*	P-value	Fold Enrichment	FDR
**Clusters A & D (regulated by MAFF)**			
TNF signaling pathway (hsa04668)	0.000	10.03	0.29
NF-kappa B signaling pathway (hsa04064)	0.012	8.15	12.31
cGMP-PKG signaling pathway (hsa04022)	0.013	5.34	13.02
Leukocyte transendothelial migration (hsa04670)	0.027	6.01	25.68
Retinol metabolism (hsa00830)	0.049	8.18	42.77
**Clusters B & E (regulated by MAFB)**			
Retinol metabolism (hsa00830)	0.001	11.07	1.05
Complement and coagulation cascades (hsa04610)	0.011	8.35	11.96
**Clusters C & F (regulated by MAFB and MAFF)**			
Adipocytokine signaling pathway (hsa04920)	0.041	8.97	37.08

*Analyzed by DAVID Bioinformatics Resources

Figure S9. KEGG pathway analysis of gene expression profile in MAFB and MAFF knockdown cells. (**A**) HuH7 cells stably transfected with shN, shMAFB, or shMAFF were treated with EtOH or 2 μM ATRA for 12 h followed by recovery of mRNA, which were subjected to microarray analysis using Illumina Human HT-12 v4 Expression BeadChip. The ratios of gene expression levels in cells treated with ATRA to those of EtOH were used for the cluster analysis. (**B**) Significantly enriched KEGG pathways in the clusters.

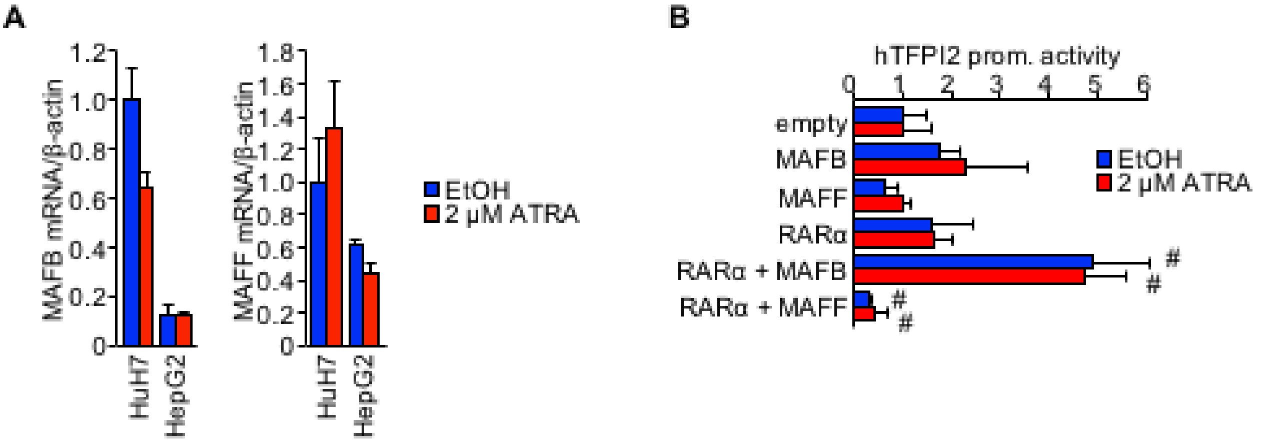

Figure S10. (**A**) MAFB and MAFF expression in HCC cell lines. The relative expression levels of MAFB (**left**) or MAFF (**right**) to β-actin in HuH7 or HepG2 cells treated with EtOH (open bars) or 2 μM ATRA (filled bars) for 12 h (n = 4). (**B**) A luciferase reporter vector driven by the human TFPI2 promoter was transfected along with pDNA expressing the indicated transcription factor genes into HepG2 cells. At 24 h post-transfection, EtOH (blue) or 2 μM ATRA (red) were added to the cells, and further incubated for 24 h, followed by dual luciferase assay (n = 4). #, $p < 0.05$ (vs. RARα) (Tukey-Kramer's test).

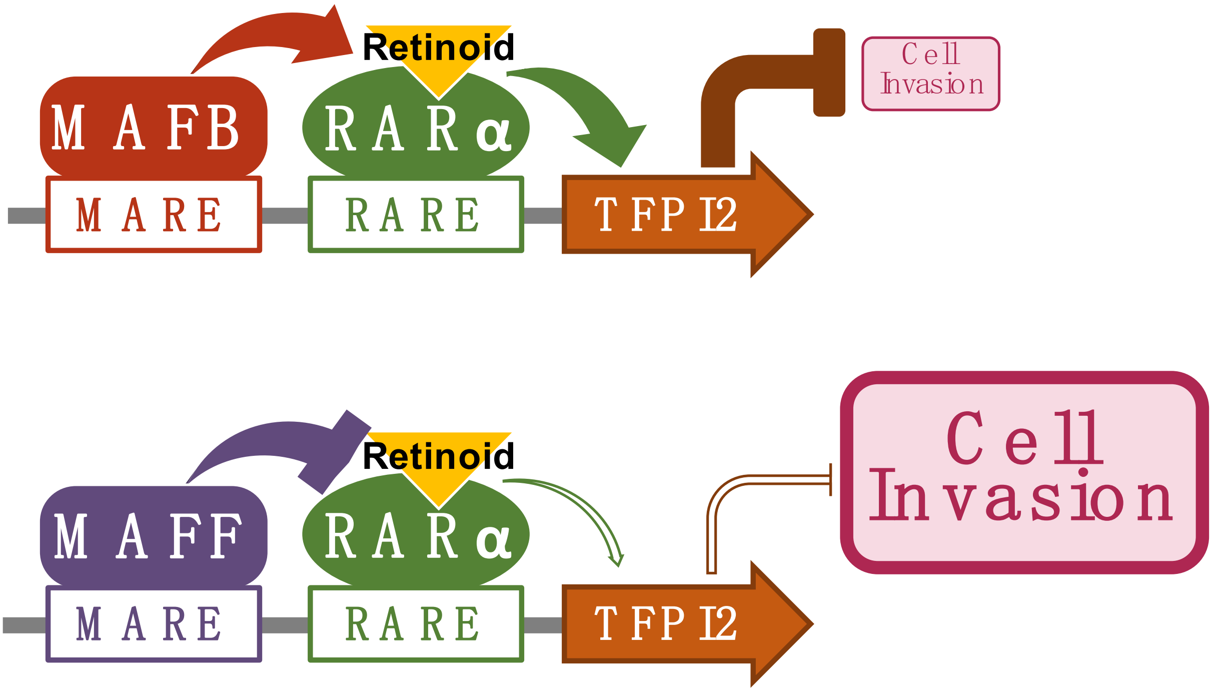

Figure S11. MAFB and MAFF were identified as factors regulating RARα activity on the TFPI2 promoter. This mechanism underlying retinoid-mediated inhibition of liver cancer progression could be promising in the therapeutic management of hepatocellular carcinoma.

Table S1. Clinical and demographic characteristics of patients.

N	44	
Male/Female	34/10	
Age (yrs) (Mean ± SD)	62.1 ± 16.7	
Normal liver	8	
Fatty liver	5	
Hepatitis	3	
Cirrhosis	5	
Hepatocellular carcinoma (W/M/P/?)	23	
TNM stage	I	5
	II	8
	IIIA	8
	IV	2
Tumor grade	G1	6
	G2	11
	G3	4
	?	2

G1, well differentiated; G2, moderately differentiated; G3, poorly differentiated; ?, not reported.

Table S2. Correlation analysis of gene expression with liver cancer features.

	**TNM Stage (*n* = 23)**	**Histologic Grade (*n* = 21)**
TFPI2	rs = 0.066<br>$p > 0.05$	rs = −0.159<br>$p > 0.05$
RARα	rs = 0.159<br>$p > 0.05$	rs = −0.576<br>$p < 0.01$
RARβ	rs = 0.291<br>$p > 0.05$	rs = 0.301<br>$p > 0.05$
MAFB	rs = −0.362<br>$p < 0.05$	rs = −0.173<br>$p > 0.05$
MAFF	rs = 0.266<br>$p > 0.05$	rs = −0.224<br>$p > 0.05$

Spearman's correlation analysis in HCC was performed between gene expression levels, and TNM stage (I, $n = 5$; II, $n = 8$; IIIA & IV, $n = 10$), or histologic grade (G1, $n = 6$; G2, $n = 11$; G3, $n = 4$). rs, Spearman's correlation coefficient.
